# Prediction models for sarcopenia in older adults in China: a scoping review

**DOI:** 10.3389/fmed.2026.1835309

**Published:** 2026-05-28

**Authors:** Kanfei Yao, Yihong Xu, Jia Xu, Xiaojie Zhang, Fanglei Gu, Lijiangshan Hua, Xiuping Li

**Affiliations:** 1 Nursing Department, Sir Run Run Shaw Hospital, Zhejiang University School of Medicine, Hangzhou, China; 2 Zhejiang Chinese Medical University, Hangzhou, China

**Keywords:** China, older adults, prediction model, sarcopenia, scoping review

## Abstract

**Purpose:**

This scoping review synthesized research on sarcopenia prediction models for older adults in China to identify key limitations constraining their clinical applicability.

**Methods:**

Adhered to the Arksey and O’Malley framework and the PRISMA-ScR guidelines for this scoping review. Sarcopenia prediction models were systematically retrieved from PubMed, Embase, Web of Science, CNKI, and Wanfang, from inception to December 31, 2024. Two reviewers independently screened the literature and extracted data. Eligible studies were narratively synthesized.

**Results:**

This review identified 20 articles encompassing 34 prediction models. The reported prevalence of sarcopenia across studies ranged from 12 to 54.17%. Logistic regression and machine learning were the predominant modeling techniques. The number of predictor variables per model ranged from 3 to 8. The most frequently included predictors were age (*n* = 24), BMI (*n* = 23), and sex (*n* = 15). The models demonstrated acceptable discriminative ability, with AUC values ranged from 0.706 to 0.974. Sensitivity ranged from 0.405 to 0.963, whereas specificity ranged from 0.400 to 0.947.

**Conclusion:**

Despite the rapid growth of sarcopenia prediction models in recent years, this review reveals persistent deficiencies in variable selection, methodological rigor, and external validation, which collectively limit their clinical applicability. Addressing these issues is essential for developing predictive tools that are statistically robust, clinically applicable, and tailored to China’s aging population.

## 1 Introduction

Sarcopenia is a progressive and generalized skeletal muscle disorder, characterized by loss of muscle mass accompanied by reduced muscle strength and/or physical performance (1). It has emerged as a significant public health challenge in the context of global population aging. Epidemiological surveys in China report a sarcopenia prevalence of 19.8%, with moderate and severe cases accounting for 11.9 and 7.9%, respectively (2). Sarcopenia is strongly associated with adverse outcomes, including increased risk of fractures, falls, disability, and mortality, thereby imposing a substantial burden on individuals, families, and healthcare systems (3, 4).

Although sarcopenia is preventable and potentially reversible, early identification remains challenging because the condition often progresses insidiously without obvious clinical manifestations in its initial stages (5, 6). Globally, the screening and diagnosis of sarcopenia continue to face persistent challenges in balancing feasibility with diagnostic accuracy. Commonly used screening tools, such as the SARC-F questionnaire and Ishii’s score, are simple and convenient for large-scale application; however, their sensitivity for detecting early-stage sarcopenia remains suboptimal across different populations and healthcare settings (7, 8). In contrast, standard diagnostic methods—including Dual-energy X-ray Absorptiometry (DXA), Bioelectrical Impedance Analysis (BIA), and Computed Tomography (CT)—provide more reliable assessments of muscle mass and function, but their routine implementation often depends on specialized equipment, trained personnel, and substantial healthcare resources. Consequently, these methods may be difficult to apply consistently in primary care, community-based practice, and resource-limited settings (9, 10). These implementation barriers are increasingly recognized as a global challenge in sarcopenia management. In response, prediction models based on routinely available clinical variables have emerged as potentially practical and scalable tools for early risk stratification and targeted intervention across diverse healthcare settings (11).

In recent years, the number of sarcopenia prediction models has grown rapidly. However, this rapid proliferation has introduced a new challenge: the methodological quality and clinical applicability of existing models remain uncertain, and clinicians often lack clear guidance regarding which model is most appropriate for specific populations and clinical settings (12). Previous systematic reviews (9, 13) have summarized the predictive performance of sarcopenia models and generally reported acceptable discriminative ability. Nevertheless, despite promising statistical performance, the real-world clinical applicability of these models remains limited. Therefore, this review systematically maps the current landscape of sarcopenia prediction models for older adults in China, with the aim of identifying factors that constrain their clinical applicability and informing future model development and validation.

## 2 Methods

### 2.1 Design and research questions

Given the substantial heterogeneity in study populations, settings, and methodologies, a scoping review was deemed more appropriate than a meta-analysis. This review was conducted in accordance with the methodological framework proposed by Arksey and O’Malley (14) and reported following the Preferred Reporting Items for Systematic Reviews and Meta-Analyses Extension for Scoping Reviews (PRISMA-ScR) guidelines (15). The protocol was registered on the Open Science Framework (https://doi.org/10.17605/OSF.IO/BEU64). The study was guided by the following research questions: (1) What is the current state of research on sarcopenia prediction models for older adults in China? (2) What methods have been used for model development and validation? and (3) What are the major limitations of existing sarcopenia prediction models for older adults in China?

### 2.2 Information sources and search strategy

A systematic literature search was conducted in PubMed, Embase, Web of Science, China National Knowledge Infrastructure (CNKI), and Wanfang, from database inception to December 31, 2024. The initial search strategy was developed for PubMed and subsequently adapted for use in the remaining databases. In addition, the reference lists of relevant studies were manually screened to supplement the electronic search. The complete search strategies for all databases are provided in Supplementary file S1.

### 2.3 Inclusion and exclusion criteria

The inclusion criteria were as follows: (1) participants were older adults (≥60 years); (2) studies focused on the development or validation of sarcopenia prediction models; (3) articles were published in English or Chinese; and (4) studies were conducted in China. Studies were excluded if they: (1) analyzed risk factors without developing a prediction model; (2) were non-peer-reviewed publications, such as conference abstracts or gray literature (i.e., documents published outside traditional peer-reviewed channels); or (3) did not have accessible full texts.

### 2.4 Study selection

Two researchers (K. Y. and J. X.) independently screened all identified records. Any discrepancies were resolved through discussion or, when necessary, consultation with a third researcher (L. H.). Duplicate records were removed automatically using EndNote X9, followed by manual verification to identify any remaining duplicates. Titles and abstracts were initially screened to exclude clearly irrelevant studies, after which the full texts of potentially eligible articles were assessed for final inclusion.

### 2.5 Data extraction

A standardized data extraction form was developed based on the Critical Appraisal and Data Extraction for Systematic Reviews of Prediction Modeling Studies (CHARMS) framework (16). Extracted information included the following: author and publication year, data source, participant characteristics, study location, diagnostic criteria, sarcopenia case/sample size, handling of missing data, modeling method, candidate predictors, variable selection procedures, calibration method, Area Under the Curve (AUC), sensitivity, specificity, validation method, final predictors, and presentation format. Two researchers (K. Y. and X. Z.) independently extracted the data, and any discrepancies were resolved through discussion, with iterative refinement of the extraction process to ensure accuracy and consistency.

## 3 Results

**Figure 1:**
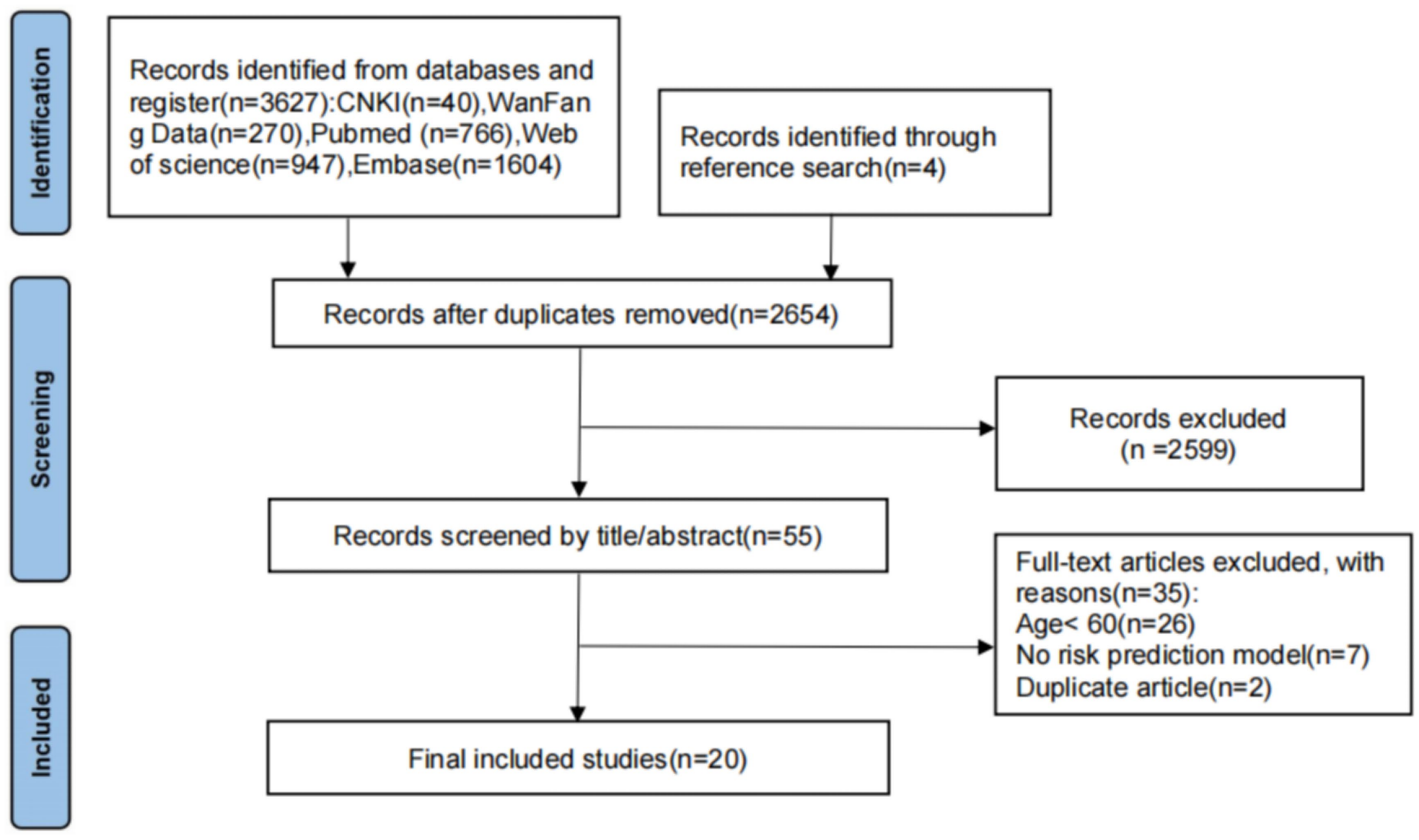
PRISMA flow diagram of the study selection process.

A total of 3,631 articles were identified through the systematic search. After duplicate removal using EndNote X9 (*n* = 977), 2,599 records were excluded following title and abstract screening. The remaining 55 articles underwent full-text assessment, resulting in the exclusion of 35 studies for the reasons detailed in Figure 1. Ultimately, 20 articles (17–36) were included in the review.

### 3.1 Study characteristics

Table 1 summarizes the basic characteristics of the 20 included studies (17–36). 16 studies (17–32) were cross-sectional, three studies (33–35) were based on retrospective data, and one study (36) used data from China Health and Retirement Longitudinal Study (CHARLS) database. The studies were conducted predominantly in hospital and community settings. Among the studies, 13 (17–19, 21, 23–26, 29, 31–34) were conducted in hospitals and involved older adults with chronic conditions, including stroke, Chronic Obstructive Pulmonary Disease (COPD), lung cancer, hip fracture, and Type 2 Diabetes Mellitus (T2DM). Three studies (17, 18, 22) used the SARC-F for sarcopenia screening, whereas the remaining 17 studies (19–21, 23–36) applied the Asian Working Group for Sarcopenia (AWGS) 2014 or 2019 consensus diagnosis criteria. Across all studies, 14,962 participants were included in model development, with the number of sarcopenia cases ranging from 20 to 1,003 and prevalence estimates ranging from 12 to 54.17%. Regarding missing data handling, 17 studies (17–25, 27, 28, 30–35) did not report missing values or describe methods for handling missing data, whereas only three studies (26, 29, 36) provided information on missing data management.

**Table 1:** Basic characteristics of the included studies (*n* = 20).

Author (Year)	Source of data	Participants	Location	Diagnostic criteria	Sarcopenia case/Sample case (%)	Missing data handling
Kong L (2024) (17)	Cross-sectional study	Older patients with stroke	The First Affiliated Hospital of Jinzhou Medical University, Liaoning	SARC-F	184/489 (37.6)	—
Han T (2022) (18)	Cross-sectional study	Older inpatients	Xinjiang Uygur Autonomous Region People’s Hospital, Urumqi	SARC-F	113/695 (16.3)	—
Zhang Y (2020) (19)	Cross-sectional study	Older inpatients	Department of General Medicine, Affiliated Hospital of Qinghai University, Xining	AWGS2014	83/268 (30.9)	—
Chen J (2023) (20)	Cross-sectional study	Community-dwelling older adults	5 communities in the old town of Xiangtan City (New Fourth Village, New Fifth Village, Quanxintang, Leigong Pond, Batang Community), Hunan	AWGS2019	87/556 (15.65)	—
Zhang Y (2023) (21)	Cross-sectional study	Hospitalized older patients	Department of Geriatrics, the First Affiliated Hospital of Xinjiang Medical University, Urumqi	AWGS	70/372 (18.82)	—
Liu Y (2022) (22)	Cross-sectional study	Older patients with chronic diseases	A community in Linghe District, Jinzhou City, Liaoning	SARC-F	145/460 (31.5)	—
Huang X (2024) (23)	cross-sectional study	Older patients with T2DM	Shangrao People’s Hospital, Jiangxi	AWGS2019	1003/3558 (28.19)	—
Zhou Y (2023) (24)	Cross-sectional study	Lung cancer patients	Huai ‘an Second People’s Hospital special needs ward, Jiangsu	AWGS2014	52/96 (54.17)	—
Deng Y (2020) (25)	Cross-sectional study	COPD patients	Department of Respiratory Medicine, The First Affiliated Hospital of Xinjiang Medical University, Urumqi	AWGS2014	43/225 (19.1)	—
Cui M (2020) (26)	Cross-sectional study	T2DM patients	Department of endocrinology and metabolism of the First Hospital of Jilin University, Changchun	AWGS2014	38/132 (28.8)	KNN
Huang SW (2023) (27)	Cross-sectional study	Community-dwelling older adults	Wuhan, Hubei	AWGS2019	116/966 (12.0)	—
Yang Y (2023) (28)	Cross-sectional study	Older adults	Shaoxing, Zhejiang	AWGS2019	125/633 (19.7)	—
Yin G (2023) (29)	Cross-sectional study	Older adults	Medical Examination Center of Zhongnan Hospital of Wuhan University	AWGS2019	55/180 (30.6)	Multiple imputation
Tseng TG (2020) (30)	Cross-sectional study	Community-dwelling older adults	Four aging cities (Pingtung County, Tainan City, Changhua County, and Miaoli County), and a total of 58 Taiwanese communities	AWGS2014	179/1025 (17.5)	—
Jiang Y (2023) (31)	Cross-sectional study	Older patients	Geriatric Medicine Department of Affiliated Kunshan Hospital of Jiangsu University	AWGS2019	151/303 (49.8)	—
Yu S (2022) (32)	Cross-sectional study	Older patients with hip fracture	The First Affiliated Hospital of Wenzhou Medical University	AWGS2019	44/212 (20.7)	—
Chen X (2023) (33)	Retrospective study	Older COPD patients	Department of Respiratory Medicine, Xinjiang Uygur Autonomous Region People’s Hospital, Urumqi	AWGS2019	52/208 (25)	—
Chen L (2023) (34)	Retrospective study	Older stroke patients	Nantong Second People’s Hospital, Jiangsu	AWGS2019	20/80 (25)	—
Mo Y (2022) (35)	Retrospective study	Community-dwelling older residents	Hunan	AWGS2019	263/1050 (25.0)	—
Li Q (2024) (36)	CHARLS2015 data	Older adults	150 counties and 450 communities (villages) within 28 provinces, autonomous regions, and municipalities nationwide	AWGS2019	997/3454 (28.8)	Multiple imputation

“—”, not reported; COPD, Chronic obstructive pulmonary disease; T2DM, Type 2 Diabetes Mellitus; AWGS, Asian Working Group for Sarcopenia; KNN, k-Nearest Neighbor.

### 3.2 Model development

**Figure 2:**
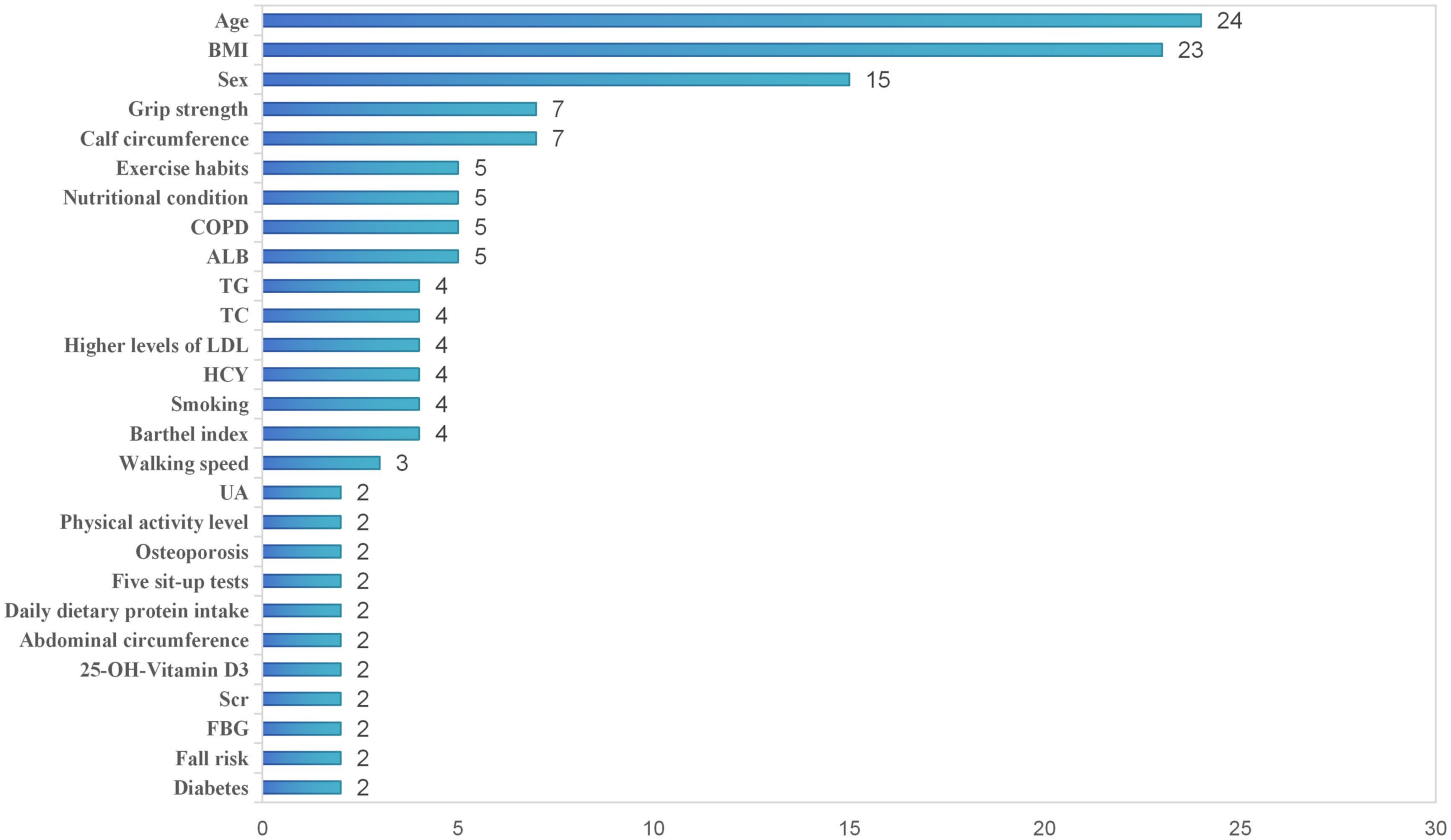
Frequency distribution of predictors appearing in at least two models. Numerical values indicate the number of models in which each predictor was included.

Table 2 summarizes the characteristics of the 34 risk prediction models derived from the 20 included studies. 12 models (35.3%) employed traditional logistic regression, seven models (20.6%) utilized R software packages, and 15 models (44.1%) applied machine learning methods. The number of candidate predictors ranged from 10 to 65. Common variable selection methods included univariable analysis, multivariable logistic regression, Least Absolute Shrinkage and Selection Operator (LASSO) regression, and factor analysis. Among these, 14 models (41.2%) used univariable analysis combined with multivariable logistic regression to determine the final predictors. Across all included models, the number of predictor variables ranged from 3 to 8. Detailed characteristics of the final predictors are presented in Table 3. The most frequently included predictor was age (*n* = 24, 70.6%), followed by Body Mass Index (BMI) (*n* = 23, 67.7%), and sex (*n* = 15, 44.1%). Figure 2 displays a histogram of predictors that appeared in at least two models to enhance readability.

**Table 2:** Information of the included prediction models (*n* = 20).

Author (Year)	Modeling method	Candidate predictors (n)	Variable selection	Calibration method	AUC	Sensitivity	Specificity	Validation method
Kong L (2024) (17)	LR; DT	23	LR	HL	Model 1: 0.959 Model 2: 0.892^A^; 0.826^B^	Model 1: 0.880 Model 2: 0.892^A^; 0.826^B^	Model 1: 0.911 Model 2: 0.939^A^; 0.846^B^	Model 1: — Model 2: C(Random split validation method)
Han T (2022) (18)	LR; DT	20	Univariable analysis	HL	Model 1: 0.864 Model 2: 0.779	—	—	—
Zhang Y (2020) (19)	R software	13	Univariable analysis and multivariable LR	—	—	—	—	C: Bootstrap sampling
Chen J (2023) (20)	RMS package of R software	33	Univariable analysis and multivariable LR	—	0.895	—	—	—
Zhang Y (2023) (21)	LR; DT; NN	41	Univariable analysis	HL	Model 1: 0.882 Model 2: 0.874 Model 3: 0.890	Model 1: 0.855 Model 2: 0.899 Model 3: 0.797	Model 1: 0.767 Model 2: 0.697 Model 3: 0.853	C: Random split validation method
Liu Y (2022) (22)	LR	11	Univariable analysis and binary LR	HL	0.955	0.897	0.883	—
Huang X (2024) (23)	RMS package of R software	16	Lasso regression and multivariable LR	HL	0.898^A^; 0.880^B^	0.854^A^; 0.826^B^	0.848^A^; 0.842^B^	C: Random split validation method D: Temporal validation
Zhou Y (2023) (24)	RMS package of R software	20	Univariable analysis and multivariable LR	HL	0.917	0.9231	0.75	—
Deng Y (2020) (25)	RMS and ROCR package of R software	10	Univariable analysis and multivariable LR	—	0.954; 0.917; 0.860	—	—	C: Random split validation method and 10-fold cross validation
Cui M (2020) (26)	SVM; RF	—	Backward selection method	—	Model 1: 0.85 Model 2: 0.87 Model 3: 0.87 Model 4: 0.76 Model 5: 0.81 Model 6: 0.85	Model 1: 0.514 Model 2: 0.595 Model 3: 0.568 Model 4: 0.405 Model 5: 0.486 Model 6: 0.432	Model 1: 0.947 Model 2: 0.947 Model 3: 0.937 Model 4: 0.937 Model 5: 0.895 Model 6: 0.916	C: K-fold cross validation and leave one out cross validation
Huang SW (2023) (27)	LR	51	Univariable analysis and multivariable LR	HL	0.930^A^; 0.897^B^	0.963^A^; 0.943^B^	0.762^A^; 0.735^B^	C: Random split validation method and bootstrap sampling
Yang Y (2023) (28)	Regplot package of R software	14	Multivariable LR with a forward stepwise method	HL	0.974^A^; 0.968^B^	—	—	C: Random split validation method
Yin G (2023) (29)	LR	49	Univariate LR and LASSO regression	CC; HL	0.90^A^; 0.92^B^	0.81^A^; 0.85^B^	0.91^A^; 0.89^B^	C: Random split validation method and bootstrap sampling
Tseng TG (2020) (30)	LR	23	Multivariable LR	—	0.757	0.718	0.711	C: Random split validation method
Jiang Y (2023) (31)	LR; SVM; KNN; XGboost	23	Univariable analysis and multivariable LR	—	Model 1: 0.764 Model 2: 0.775 Model 3: 0.748 Model 4: 0.706	Model 1: 0.686 Model 2: 0.657 Model 3: 0.657 Model 4: 0.914	Model 1: 0.800 Model 2: 0.840 Model 3: 0.740 Model 4: 0.400	C: Random split validation method
Yu S (2022) (32)	LR	14	Factor analysis	—	0.836^B^	0.769^B^	0.902^B^	C: Random split validation method
Chen X (2023) (33)	LR	17	Univariable analysis and multivariable LR	HL	0.756^A^; 0.808^B^	0.808^A^; 0.833^B^	0.632^A^; 0.702^B^	D: Temporal validation
Chen L (2023) (34)	LR	13	Univariable analysis and multivariable LR	HL	0.826	0.8471	0.7988	—
Mo Y (2022) (35)	LR	11	Univariable analysis and multivariable LR	HL	0.827^A^; 0.755^B^	0.681^A^; 0.679^B^	0.825^A^; 0.758^B^	C: Random split validation method and cross validation
Li Q (2024) (36)	LR	65	LASSO regression	CC; HL	0.77^A^; 0.76^B^	—	—	C: Random split validation method

“—,” not reported; A, development cohort; B, validation cohort; C, internal validation; D, external validation; LR, Logistic Regression; DT, Decision Tree; NN, Neural Network; SVM, Support Vector Machine; RF, Random Forest; KNN, k-Nearest Neighbor; XGboost, eXtreme Gradient Boosting; CC, Calibration Curve; HL, Hosmer-Lemeshow test.

**Table 3:** Final predictor of the included prediction models (*n* = 20).

Author (Year)	Final Predictors	Presentation
Kong L (2024) (17)	Model 1: smoking, age, ADL, fall risk, nutrition, and exercise habits Model 2: smoking, age, ADL, nutrition, and exercise habits	Model 1: Nomogram Model 2: DT
Han T (2022) (18)	Model 1: combined with chronic bronchitis, combined with osteoporosis, BI, and five sit-up tests Model 2: BI, stand up-walk timing test, and five sit-up tests	Model 1: — Model 2: DT
Zhang Y (2020) (19)	Age, BMI, osteoporosis, and smoking	Nomogram
Chen J (2023) (20)	Age, BMI, thigh circumference, and calf circumference	Nomogram
Zhang Y (2023) (21)	Model 1: sex, BMI, walking speed, grip strength, and abdominal circumference Model 2: BMI, nutritional risk, walking speed, sex, and ALB Model 3: BMI, grip strength, walking speed, abdominal circumference, and age	Model 1: Formula of risk score obtained by regression coefficient of each factor Model 2: DT Model 3: NN
Liu Y (2022) (22)	Age, exercise habits, number of illnesses, malnutrition, risk of falling, and fatigue	Nomogram
Huang X (2024) (23)	Age, duration of diabetes, FBG, 2 h PBG, and diabetic complications	Nomogram
Zhou Y (2023) (24)	Long-term smoking history, NRS2002 score, and BMI	Nomogram
Deng Y (2020) (25)	Model 1, 2, and 3: daily dietary protein intake, physical activity level, and COPD stages	Nomogram
Cui M (2020) (26)	Model 1 and 4: age, sex, and BMI; Model 2 and 5: age, sex, BMI, grip strength, and calf circumference; Model 3 and 6: age, sex, BMI, grip strength, calf circumference, serum albumin, and 25-OH-Vitamin D3	—
Huang SW (2023) (27)	Age, BMI, calf circumference, congestive heart failure, and COPD	Nomogram
Yang Y (2023) (28)	BMI, age, UA, ALT, and sex	Nomogram
Yin G (2023) (29)	Age, ALB, blood urea nitrogen, grip strength, and calf circumference	Nomogram
Tseng TG (2020) (30)	Female, age, receiving social assistance pension, absence of exercise, underweight, abnormal fasting glucose level, and abnormal creatinine level	—
Jiang Y (2023) (31)	Model 1, 2, 3, and 4: older age, male sex, lower levels of BMI, TC, TG, higher levels of LDL, and HCY	—
Yu S (2022) (32)	Scr, CysC, CysC-based eGFR, UA, SI, new SI, Hb and ALB	Formula of risk score obtained by regression coefficient
Chen X (2023) (33)	Age, BMI, number of acute exacerbations, high CAT score, longer COPD course, and high FEV l/FVC%	Formula of risk score obtained by regression coefficient of each factor
Chen L (2023) (34)	Age, BMI, nutritional condition, stroke, bone density, and hyperlipidemia	Formula of risk score obtained by regression coefficient of each factor
Mo Y (2022) (35)	Age, BMI, marital status, regular physical activity habit, uninterrupted sedentary time, and dietary diversity score	Nomogram
Li Q (2024) (36)	Sex, BMI, MSBP, MDBP, and pain	Nomogram

“—,” not reported; ADL, Activity of daily living; BI, Barthel index; BMI, Body mass index; COPD, Chronic obstructive pulmonary disease; ALB, Albumin; FBG, Fasting blood glucose; 2 h PBG, 2-hour postprandial blood glucose; MSBP, Mean Systolic Blood Pressure; MDBP, Mean Diastolic Blood Pressure; UA, uric acid; ALT, Alanine Aminotransferase; TC, Total Cholesterol; TG, Triglyceride; LDL, Low-density Lipoprotein; HCY, homocysteine; Scr, Serum Creatinine; CysC, serum cystatin C; CysC-based eGFR, estimated glomerular filtration rate based on cystatin C; Hb, Haemoglobin; SI, sarcopenia index.

### 3.3 Model validation

Common internal validation methods included random split validation, cross-validation, and bootstrap sampling. Among the 34 models, 12 (35.3%) applied random split validation only, six (17.6%) incorporated cross-validation and bootstrap sampling in addition to random split validation, one (2.9%) used bootstrap sampling alone, and six (17.6%) employed both K-fold cross-validation and leave-one-out cross-validation. Only two models (5.8%) underwent external validation, with one using a temporally distinct dataset and the other incorporating both internal and external validation. Notably, seven models (20.6%) did not report any form of validation.

Model performance was evaluated in terms of discrimination, sensitivity, specificity, and calibration. Discrimination was primarily quantified using the AUC/C-statistics. All models except that of Zhang et al. (19) reported AUC values, which ranged from 0.706 to 0.974. Sensitivity ranged from 0.405 to 0.963, whereas specificity ranged from 0.400 to 0.947. Regarding calibration, 11 models (32.4%) used the Hosmer-Lemeshow test, all demonstrating acceptable fit (*p* > 0.05). Two models (5.9%) combined Hosmer-Lemeshow test with calibration plots, whereas 21 models (61.8%) did not report any calibration assessment.

Regarding model presentation, nomograms were the most common format, accounting for 14 models (41.2%), including three nomogram models developed by Deng et al. (25) Regression equations were used in four models (11.8%), decision trees in three models (8.8%), and neural network representations in one model (2.9%). The remaining models did not specify a presentation format.

## 4 Discussion

Among the included studies, the reported prevalence of sarcopenia ranged from 12 to 54.17%. Logistic regression and machine learning were the predominant modeling techniques, with age, BMI, and sex emerging as the most frequently included predictors. Overall, the models demonstrated acceptable discriminative ability (AUC: 0.706–0.974), whereas the sensitivity and specificity ranged widely (0.405–0.963 and 0.400–0.947, respectively). The value of this review lies not only in delineating the current landscape of sarcopenia prediction model research in China, but also in illuminating the key challenges and opportunities associated with the field’s transition from rapid expansion to methodological refinement.

Among the included models, a total of 53 predictors were identified, of which only 16 (30.2%) appeared in three or more studies, whereas many predictors were reported in only a single study. This heterogeneity in variable selection reduces comparability among models and hinders the establishment of a unified predictor set, thereby limiting reproducibility and generalizability. Two major factors contribute to this issue. First, variable selection is often constrained by data availability. Existing studies predominantly rely on structured data readily accessible from electronic medical records, including demographic, laboratory, and functional parameters, consistent with previous findings (37). Consequently, potentially important predictors such as dietary habits, physical activity, and psychosocial factors—are frequently omitted, not due to lack of clinical relevance, but because their collection requires additional effort. Second, methodological limitations in variable selection remain common. Most studies employed univariable analysis followed by multivariable logistic regression, prioritizing statistical significance while neglecting complex interrelationships among predictors. As a result, clinically relevant predictors with non-significant *p*-values may be excluded, leading to predictor sets with limited stability and reproducibility (38).

Unreported handling of missing represents another important source of methodological instability (39). In this review, only three studies described their methods for handling missing data, whereas the remaining studies did not report any approach. This may lead to bias in model fitting and instability in model parameters, ultimately affecting the predictive accuracy (37). Regarding model development, logistic regression and machine learning were the most frequently employed techniques, reflecting researchers’ trade-off between model interpretability and predictive performance. Although logistic regression models are widely used, their reliance on linear assumptions limits their ability to account for complex interactions among factors, thus reducing predictive accuracy (40). In contrast, machine learning models are better equipped to capture the complex nonlinear relationships among age, nutritional indicators, and comorbidities; however, their “black-box” nature may hinder implementation in clinical settings that require transparent decision-making processes (41). Therefore, model selection should prioritize clinical applicability rather than excessive technological complexity, with greater emphasis placed on addressing the practical needs of real-world clinical settings.

In practical settings such as primary care, rapidly interpretable tools, including nomograms or risk scores, may offer greater clinical utility than complex ensemble algorithms (12). In this review, 41.2% of models did not report a user-facing presentation format, which may hinder their clinical translation. Therefore, promoting the development of visual tools (e.g., nomograms) (42), embedded clinical decision support systems (43), or mobile applications (44) may represent an important strategy for improving model usability. In addition, model validation remains a major methodological challenge. Only one study implemented both internal and external validation, whereas most relied exclusively on internal validation. External validation is essential for detecting overfitting and identifying biases inherent in internal validation processes (45). The lack of external validation limits understanding of model generalizability across different populations and clinical settings (46). Future research should prioritize rigorous external validation, including the use of geographically and temporally distinct cohorts, to ensure broader applicability and robustness of prediction models in real-world clinical practice.

Substantial variation in sarcopenia prevalence (12–54.17%) was observed across the included studies. This variation is not solely attributable to the heterogeneity of the Chinese population, but rather reflects the combined influence of multiple factors. Although most studies applied the formal diagnostic criteria established by the AWGS, a subset utilized screening tools (e.g., the SARC-F questionnaire) to define prevalence. Given the inherent limitations of screening tools in terms of sensitivity and specificity, the true prevalence may be obscured (47). Furthermore, one study (36) based on the CHARLS 2015 database applied the AWGS 2019 criteria for retrospective diagnosis. This temporal mismatch between diagnostic criteria and data collection may introduced biased in historical prevalence estimates (48). More importantly, differences in sarcopenia type across study populations further contributed to the wide variation in prevalence estimates. Among the included studies, older adults with chronic conditions (e.g., stroke, COPD, T2DM, cancer, hip fracture) exhibited higher prevalence estimates (16.3–54.17%) than community-based studies involving relatively healthy older adults (12.0–25.0%). Some community-based studies did not specify whether participants with chronic comorbidities were excluded, potentially introducing heterogeneity due to the inclusion of both primary and secondary sarcopenia cases. This variation is not merely attributable to differences in diagnostic criteria or sample sources, but is also closely related to the underlying pathophysiology of sarcopenia (age-driven versus disease-driven) (9). These findings highlight the importance of clearly defining population characteristics when reporting prevalence estimates to minimize potential bias. Accordingly, promoting the standardized application of diagnostic criteria and improving sample representativeness are essential for enhancing the methodological rigor of future sarcopenia prediction research.

## 5 Limitations

This study has several limitations. First, the literature search was restricted to publications in Chinese and English, which may have resulted in the omission of relevant studies published in other languages. However, because this review focuses specifically on the Chinese population, this language restriction primarily reflects the geographical and cultural scope of the study rather than a methodological limitation. While it is possible that relevant studies exist in other languages, these are expected to be limited. Second, no formal quality assessment was conducted for the included studies, which limits our ability to evaluate the methodological rigor and potential biases. Third, most included models were developed using cross-sectional or retrospective data, with only a limited number derived from prospective cohort studies. Consequently, the long-term predictive value of these models for future sarcopenia risk remains uncertain and requires further validation.

## 6 Conclusion

This review identified 20 articles encompassing 34 prediction models, thereby systematically mapping the current landscape of sarcopenia prediction model research for older adults in China. Although the number of sarcopenia prediction models has increased rapidly in recent years, persistent limitations remain in variable selection, methodological rigor, and external validation, collectively constraining their clinical applicability. Future research should prioritize clearly defining the intended use and application scenarios of prediction models, selecting clinically relevant and modifiable predictors, and conducting rigorous external validation. These efforts are essential for developing predictive tools that are methodologically robust, clinically applicable, and tailored to the needs of China’s aging population. Ultimately, improving the quality and applicability of these models may facilitate early risk stratification and support evidence-based clinical decision-making in diverse healthcare settings.

## Data Availability

The datasets analyzed in this review are derived from previously published studies, which are publicly available through academic databases (PubMed, Embase, Web of Science, CNKI, and Wanfang). A detailed search strategy and list of included studies are provided in the manuscript and Supplementary materials.
