# Supplementary material for: Prediction models for sarcopenia in older adults in China: a scoping review
Source: Front Med (Lausanne). 2026 May 28;13:1835309. doi: 10.3389/fmed.2026.1835309 (PMC13253269; doi:10.3389/fmed.2026.1835309)
Supplement: Supplementary file 1 [file Data_Sheet_1.pdf]

S1 File. Search strategy for all databases

Pubmed

#1 ("sarcopeni*"[Title/Abstract] OR "muscle mass"[Title/Abstract] OR "muscle strength"[Title/Abstract] OR "hand strength"[Title/Abstract] OR "grip strength"[Title/Abstract] OR "muscle atrophy"[Title/Abstract] OR "muscle wasting"[Title/Abstract] OR "Sarcopenia"[Mesh])

#2 ("risk assess*"[Title/Abstract] OR "risk screen*"[Title/Abstract] OR "risk scor*"[Title/Abstract] OR "risk identif*"[Title/Abstract] OR "risk detect*"[Title/Abstract] OR "risk predict*"[Title/Abstract] OR "early diagnos*"[Title/Abstract] OR "risk categor*"[Title/Abstract] OR "risk stratif*"[Title/Abstract])

#3 ("tool"[Title/Abstract] OR "rule"[Title/Abstract] OR "algorithm"[Title/Abstract] OR "score*"[Title/Abstract])

#4 #1 AND #2 AND #3 AND (1993:2024[pdat])

Embase

#1 'sarcopeni*' OR 'muscle mass' OR 'muscle strength' OR 'hand strength' OR 'grip strength' OR 'muscle atrophy' OR 'muscle wasting'

#2 'risk assess*' OR 'risk screen*' OR 'risk scor*' OR 'risk identif*' OR 'risk detect*' OR 'risk predict*' OR 'early diagnos*' OR 'risk categor*' OR 'risk stratif*'

#3 'tool' OR 'rule' OR 'algorithm' OR 'score*'

#4 #1 AND #2 AND #3 AND [article]/lim AND [english]/lim AND [embase]/lim AND [1996-2024]/py

Web of science

#1 TS=("sarcopeni*" OR "muscle mass" OR "muscle strength" OR "hand strength" OR "grip strength" OR "muscle atrophy" OR "muscle wasting")

#2 TS=("risk assess*" OR "risk screen*" OR "risk scor*" OR "risk identif*" OR "risk detect*" OR "risk predict*" OR "early diagnos*" OR "risk categor*" OR "risk stratif*")

#3 TS=("tool" OR "rule" OR "algorithm" OR "score*")

#4 PY=(1985-2024)

#5 #1 AND #2 AND #3 AND #4

CNKI

#1 肌少症 + 肌肉减少症

#2 风险预测 + 风险评估 + 风险因素

#3 模型 + 工具

#4 #1 AND #2 AND #3

资源范围：总库; 中英文扩展; 时间范围：发表时间：截至到 2024-12-31;更新时间：不限;

WanFang Data

#1 肌少症 OR 肌肉减少症

#2 风险预测 OR 风险评估 OR 风险因素

#3 模型 OR 工具

#4 #1 AND #2 AND #3

(主题:(肌少症 or 肌肉减少症) and 主题:(风险预测 or 风险评估 or 风险因素) and 主题:(模型 or 工具)) and 出版时间:[* TO 2024-12-31}
